# Genetic Analysis and Transfer of Favorable Exotic QTL Alleles for Grain Yield Across D Genome Using Two Advanced Backcross Wheat Populations

**DOI:** 10.3389/fpls.2019.00711

**Published:** 2019-06-04

**Authors:** Ali Ahmad Naz, Said Dadshani, Agim Ballvora, Klaus Pillen, Jens Léon

**Affiliations:** ^1^Institute of Crop Science and Resource Conservation, Plant Breeding, University of Bonn, Bonn, Germany; ^2^Institute of Agricultural and Nutritional Sciences, Plant Breeding, Martin Luther University of Halle-Wittenberg, Halle, Germany

**Keywords:** synthetic wheat, *Aegilops tauschii*, D genome, grain yield, exotic allele

## Abstract

Hexaploid wheat evolved through a spontaneous hybridization of tetraploid wheat (*Triticum turgidum*, AABB) with diploid wild grass (*Aegilops tauschii*, DD). Recent genome sequencing found alarmingly low genetic diversity and abundance of repeated sequences across D genome as compared to AB genomes. This characteristic feature of D genome often results in a low recombination rate and abrupt changes in chromosome, which are the major hurdles to utilize the genetic potential of D genome in wheat breeding. In the present study, we evaluated two advanced backcross populations designated as B22 (250 BC_2_F_3:6_ lines) and Z86 (150 BC_2_F_3:6_ lines) to test their yield potential and to enrich the D genome diversity simultaneously. The populations B22 and Z86 were derived by crossing winter wheat cultivars Batis and Zentos with synthetic hexaploid wheat accessions Syn022L and Syn086L, respectively. These populations were genotyped using SNP markers and phenotyped for yield traits in ten environments in Germany. Marker analysis identified lower recombination rate across D genome as compared to A and B genomes in both populations. Further, we compared the genotype data with the trait grain yield to identify favorable exotic introgressions from synthetic wheat accessions. QTL analysis identified seven and 13 favorable exotic QTL alleles associated with enhancement or at least stable grain yield in populations B22 and Z86, respectively. These favorable introgressions were located on all chromosomes from 1D to 7D. The strongest exotic QTL allele on chromosome 1D at SNP marker RAC875_c51493_471 resulted in a relative increase of 8.6% in grain yield as compared to cultivated allele. The identified exotic introgressions will help to refine useful exotic chromosome segments for their incorporation for improving yield and increasing D genome diversity among cultivated varieties.

## Introduction

Bread wheat (*Triticum aestivum* L.) is a major cereal crop utilized as staple food worldwide. The production statistic of last decade showed a sign of yield stagnancy, which is posing a serious threat to the food security for exponentially growing human population (FAO, 2017). A primary reason behind this may lie in low genetic diversity due to the bottlenecks of domestication and intensive selection within the cultivated gene-pool (Tanksley and McCouch, 1997). Narrow genetic diversity does not simply reduce the number of useful alleles, but it can cause second order genetic dilemmas like low genetic recombination especially in self-pollinating crops like wheat. This scenario thus demands efforts to harness new genetic resources for improving yield potential as well as for broadening the genetic diversity of the cultivated wheat varieties.

Hexaploid wheat is allopolyploid (2n = 6x = 42; AABBDD) evolved through a spontaneous hybridization of tetraploid wheat (AABB) with *Aegilops tauschii* (DD). More than half a century ago, the pioneering work of Kihara (1944), Sears (1944), and McFadden and Sears (1946) found (syn. *A. squarrosa*) as the progenitor of D genome of the hexaploid wheat. Since that time valuable research was made to investigate the genetic diversity and genome divergence between the domesticated and undomesticated wheat species. For instance, Lagudah and Halloran (1988) made a phylogenetic relationship among *A. tauschii* population and found highly polymorphic gliadin (*Gli-1*) locus that provides fingerprint haplotypes for a given genotype. Similarly, glutenin (*Glu-1*) locus was studied comprehensively at gene and protein levels (Payne and Lawrence, 1983; Payne, 1987; Lagudah and Halloran, 1988; Anderson et al., 1989, 2003). These reports also revealed higher allelic variation of *Glu-D1* locus in *A. tauschii* as compared to *Glu-D1* locus in hexaploid wheat. Chantret et al. (2005) studied hardness (*Ha*) locus in polyploidy and diploid wheat species to investigate the pre- and post-polyploidization evolutionary differentiation of A, B, and D. They found the loss of puroindoline a and b (*Pina* and *Pinb*) genes from the hexaploid wheat as well as a 29 kb smaller *Ha* locus in the D genome of hexaploid wheat as compared to D genome of its diploid progenitor *A. tauschii*, putatively caused by illegitimate recombination. Using whole genome sequencing, Luo et al. (2017) made a comprehensive analysis of D genome in *A. tauschii* ssp. *strangulata* accession AL8/78. This analysis found more number of annotated genes in the progenitor of the wheat D genome *A. tauschii* as compared to the D genome of wheat cultivar Chinese Spring. These reports suggest that the wheat progenitor *A. tauschii* reveals rich genetic diversity due to its wide geographical distribution, but a limited lineage of *A. tauschii* was involved in the establishment of hexaploid wheat.

The recent whole genome sequencing of hexaploid wheat revealed an in-depth insight on the genetic potential of A, B, and D genomes and their linkages among each other. Till this end, around 35.345, 35.643, and 34.212 high confidence gene have been reported across the A, B, and D genomes, respectively (IWGSC, 2018). These data suggest almost an equal distribution of genes across the individual genomes. Although, the numbers of genes are not dramatically different across the A, B, and D genome of cultivated variety Chinese Spring, an alarmingly low genetic diversity and abundance of repeated sequences across D genome are reported as compared to A and B. This characteristic feature of D genome often results in low recombination rate and abrupt changes in chromosomes, which seem to be the major hurdles to utilize the genetic potential of D genome in wheat breeding. This scenario demands the addition of new genetic resources across the D genome of hexaploid wheat to improve the recombination rate and allelic variation simultaneously. Although, *A. tauschii* population’s diversity and its implication on improving yield and yield related traits were highlighted in the past (Ogbonnaya et al., 2003, 2005), its utility and focus remained largely on the incorporation of useful alleles for improving biotic and abiotic stress tolerance traits among the cultivated wheat varieties.

In the present study, we employed an advanced backcross QTL analysis strategy to identify genome wide marker defined introgressions of *A. tauschii* associated to improving yield using a population of 400 BC_2_F_3_ lines (Kunert et al., 2007). A direct utility of the *A. tauschii* diversity and favorable alleles for improving yield in bread wheat seem not feasible because of the linkage drag of additional exotic chromosomal segments on essential breeding traits. The advanced backcross populations were established by crossing winter wheat cultivars Batis and Zentos with synthetic hexaploid wheat accessions Syn022L and Syn086L, respectively.

## Results

### SNP Markers and Their Distribution Across A, B, and D Genomes

**FIGURE 1:**
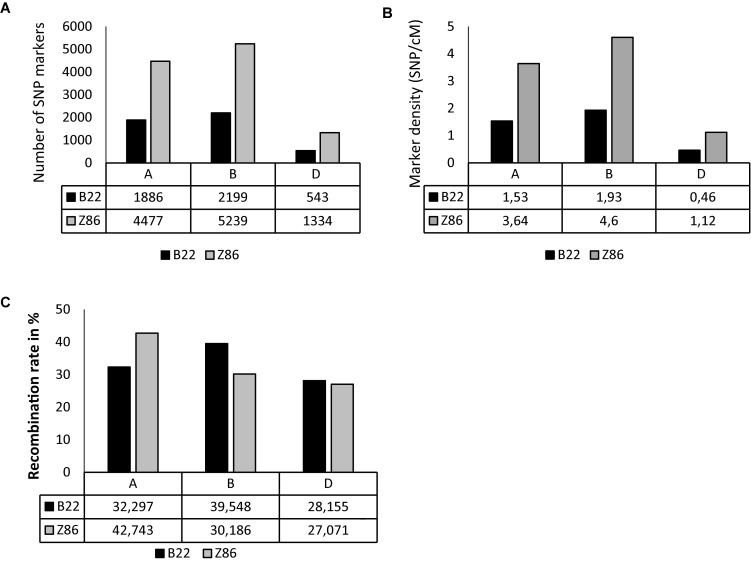
SNP genotyping. SNP marker number **(A)**, density per cM **(B)**, and recombination rate in % **(C)** across A, B, and D genomes in population B22 and Z86.

The populations B22 and Z86 were genotyped using 15 and 90 k SNP-chip arrays, respectively. Overall, the highest numbers of SNP markers and marker density were found across B genome whereas the lowest numbers of SNP markers were found across D genome in both populations (Figure 1A,B). The population Z86 revealed remarkably higher numbers of SNP markers across A (4477), B (5239), and D (1334) genomes as compared to the population B22 (Figure 1A). The lowest marker density (0.46 marker per cM) was found across the D genome especially in the population B22 (Figure 1B). The percent recombination rate was lower across the D genome as compared to A and B in the population B22 and Z86 (Figure 1C).

### Identification of Favorable Exotic QTL Allele for Grain Yield

#### Population B22

Marker by trait analysis revealed significant trait variation between the cultivated and synthetic alleles at seven SNP markers related to grain yield in population B22. These QTL effects were located on most chromosomes except on 5D. At the associated SNP loci, the relative increase in yield due to the introgression of the exotic alleles from synthetic wheat accession Syn022L, ranged from 2 to 4.6% (Table 1). The strongest exotic QTL allele was associated with SNP marker wsnp_CAP7_c1735_859875 on chromosome 6D at position 122.3 cM. Two additional exotic QTL allele that resulted in 3% increase in yield were localized on chromosomes 2D (SNP: Excalibur_c7366_1475 136.4 cM) and 3D (SNP: Excalibur_c63483_991 3.2 cM), respectively.

**Table 1:** List of SNP markers associated to grain yield variation in the population B22.

Marker^1^	Chr^2^	Pos^3^	*P*-value^4^	FDR^5^	(CC)^6^	(EE)^7^	% RP (EE)^8^
BS00022654_51	1D	135.8	<0.001	^∗∗^	74.5	76.6	2.8
Excalibur_c7366_1475	2D	136.4	<0.001	^∗∗∗^	74.4	76.7	3.1
Excalibur_c63483_991	3D	3.2	<0.001	^∗∗∗^	74.4	76.8	3.2
BobWhite_c20689_427	4D	78.6	<0.001	^∗∗∗^	74.4	76.0	2.1
BobWhite_c14066_403	6D	56.6	<0.001	^∗∗∗^	74.4	76.0	2.0
wsnp_CAP7_c1735_859875	6D	122.3	<0.001	^∗∗∗^	74.5	77.9	4.6
RAC875_c53629_483	7D	101.8	<0.001	^∗∗^	74.5	76.0	2.0


*^*1*^Significant SNP markers at the QTL effect selected by the highest *F*-value. ^*2*^Chromosomal localization of the QTL effect. ^*3*^Position of the listed marker in cM. ^*4*^Probability of significance (*P*-value). ^*5*^False discovery rate (FDR), ^∗∗^<0.01 and ^∗∗∗^<0.001. ^*6*^Least square means (lsmeans) of trait values of all investigated BC_*2*_F_*3*_ lines carrying the homozygous cultivated allele (CC) at the given marker locus. ^*7*^Lsmeans of trait values of investigated BC_*2*_F_*3*_ lines carrying the homozygous exotic (EE) allele at the given marker locus. ^*8*^Relative performance of the exotic allele at the given marker locus calculated by: [(EE)-(CC)]^∗^100/(CC), where (EE) and (CC) are lsmeans of the homozygous synthetic (exotic) and the homozygous cultivated alleles at a given marker locus.*

#### Population Z86

In population Z86, 13 exotic QTL alleles showed improved relative performance for yield in comparison to cultivated alleles from cultivar Zentos. These QTL effects were located across the 1D–7D chromosomes (Table 2). The highest numbers of QTL effects were found on chromosome 7D where four exotic QTL alleles resulted in the improvement of yield as compared to cultivated alleles. The strongest QTL effects were associated with SNP markers RAC875_c51493_471 and RAC875_c20675_268 on chromosome 1D (162.3 cM) and 3D (148.4 cM), respectively. At these loci the introgression of exotic allele from synthetic wheat accession Syn086L accounted for more than 8% increase in yield. Similarly, the introgression of exotic alleles at SNP markers Kukri_c2408_784 (1D: 3.5 cM) and BS00010664_51 (2D: 103.3 cM) showed 6.1 and 6.4% increase in yield than the corresponding cultivated alleles, respectively. On chromosome 5D at SNP BS00011794_51 locus the introgression of exotic allele resulted in 5.7% enhance of yield. Also, an exotic QTL allele of almost similar magnitude was identified on chromosome 3D at SNP marker D_contig14424_524 (121.8 cM) that accounted for 4.9% increase in yield relative to cultivated allele from Zentos.

**Table 2:** List of SNP markers associated to grain yield variation in the population Z86.

Marker^1^	Chr^2^	Pos^3^	*P*-value^4^	FDR^5^	(CC)^6^	(EE)^7^	% RP (EE)^8^
Kukri_c2408_784	1D	3.5	<0.001	^∗∗∗^	73.9	78.4	6.1
BS00063907_51	1D	115.6	<0.001	^∗∗∗^	74.9	75.4	0.6
RAC875_c51493_471	1D	162.3	<0.001	^∗∗∗^	74.1	80.4	8.6
BS00010664_51	2D	103.3	<0.001	^∗∗∗^	74.4	79.2	6.4
D_contig14424_524	3D	121.8	<0.001	^∗∗∗^	74.4	78.1	4.9
RAC875_c20675_268	3D	148.4	<0.001	^∗∗∗^	74.3	80.4	8.2
Kukri_c29969_543	5D	60.6	<0.001	^∗∗∗^	74.9	75.5	0.8
D_contig14133_180	5D	134.3	<0.001	^∗∗∗^	74.9	75.5	0.8
BS00011794_51	5D	194.9	<0.001	^∗∗∗^	74.6	78.9	5.7
Kukri_c57006_127	6D	107.4	<0.001	^∗∗∗^	74.2	77.4	4.3
BS00068485_51	7D	26.2	<0.001	^∗∗∗^	74.5	76.7	2.9
CAP7_c9278_185	7D	123.5	<0.001	^∗∗∗^	74.9	76.3	1.9
D_GA8KES401CTZ29_94	7D	161.1	<0.001	^∗∗∗^	74.6	76.6	2.7
D_F5XZDLF01CK3P1_55	7D	214.8	<0.001	^∗∗∗^	74.3	76.6	3.0


*^*1*^Significant SNP markers at the QTL effect selected by the highest *F*-value. ^*2*^Chromosomal localization of the QTL effect. ^*3*^Position of the listed marker in cM. ^*4*^Probability of significance (*P*-value). ^*5*^False discovery rate (FDR), significance level ^∗∗∗^≤0.001. ^*6*^Least square means (lsmeans) of trait values of all investigated BC_*2*_F_*3*_ lines carrying the homozygous cultivated allele (CC) at the given marker locus. ^*7*^Lsmeans of trait values of investigated BC_*2*_F_*3*_ lines carrying the homozygous exotic (EE) allele at the given marker locus. ^*8*^Relative performance of the exotic allele at the given marker locus calculated by: [(EE)-(CC)]^∗^100/(CC), where (EE) and (CC) lsmeans of the homozygous synthetic (exotic) and the homozygous cultivated alleles at a given marker locus.*

### Chromosomal Localization of Favorable Exotic Alleles for Grain Yield Across D Genome

**FIGURE 2:**
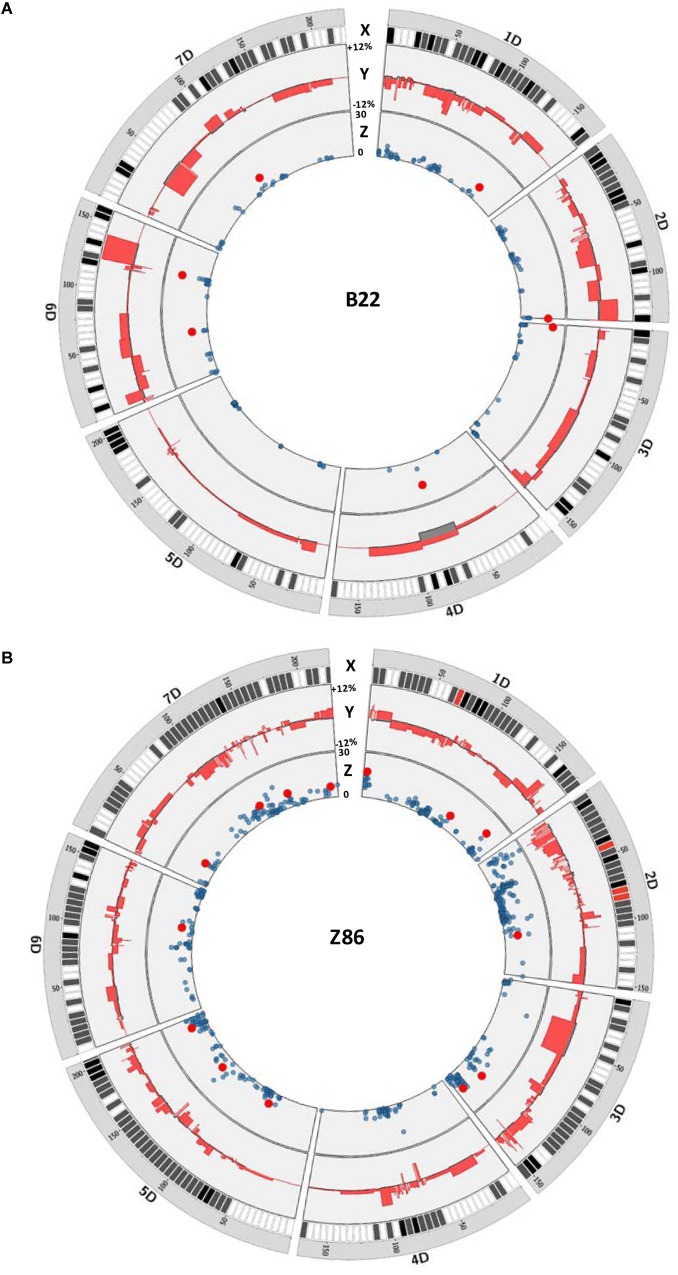
QTL map for grain yield across the D genome in population B22 **(A)** and Z86 **(B)**. In each plot X circle represents the chromosome, distribution, and density of SNP markers on the D genome from white (low density) to red (high density), Y circle illustrate the positive and negative effects of exotic alleles on grain yield relative to the population mean as reference line. The values +12 and –12% represented the positive and negative effect of exotic and cultivated alleles relative to the population mean as reference line, respectively, and Z circle shows a Manhattan plot from QTL analysis of grain yield showing significant QTL effects highlighted as red circles. The scale 0–30 represents the logarithm of odd (LOD) score.

We have plotted the marker effects across the D genome to show the length and effect of the exotic QTL alleles for grain yield in the population B22 and Z86. For this, we compared the effect of each cultivated and exotic allele with the mean grain yield of the population B22 and Z86 as base line to show relative increase and decrease in grain yield associated to exotic and cultivated alleles across the D genome (Figure 2). This analysis in the population B22 showed that all chromosomes carried exotic QTL alleles which showed both positive and negative effects on grain yield (Figure 2A). Although, exotic QTL alleles revealed variation on all chromosomes, but no significant QTL effect was found on chromosome 5D in the population B22. Surprisingly, on chromosome 4D a region of cultivated allele was associated with decrease in yield. The chromosome 6D revealed the maximum number of positive exotic QTL alleles for grain yield. Among these, the exotic introgression on the long arm of 6D showed the highest increase where the strongest exotic QTL allele was detected for yield. Similarly, the population Z86 carried favorable exotic QTL alleles for yield from chromosome 1D–7D (Figure 2B). Comparatively higher numbers of exotic QTL alleles of relatively smaller intervals were identified in the population Z86 as compared to the population B22. The exotic QTL allele accounting for the highest increase in yield was identified on chromosome 1D. At this locus at the SNP marker RAC875_c51493_471 the exotic allele revealed the highest increase in grain yield.

### Distribution and Effects of Four Exotic QTL Alleles in Population B22 and Z86

**FIGURE 3:**
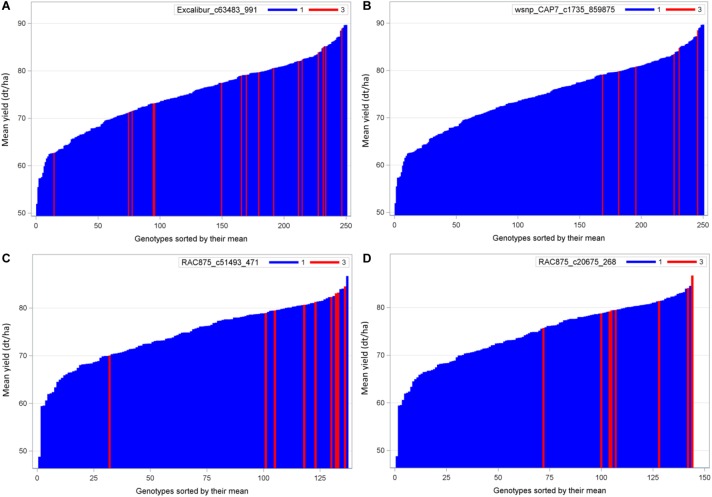
Needle plot of two strongest favorable exotic QTL alleles for yield in each population B22 **(A,B)** and Z86 **(C,D)**. Blue and red bars on *x*-axis represent the BC_2_F_3_ lines carrying homozygous cultivated (CC = 1) and exotic (EE = 3) alleles, respectively. The *y*-axis showed the grain yield in desi tons per hectare (dt/ha).

QTL analysis identified 20 QTL where the performance of exotic alleles was higher or at least comparable to the cultivated allele in the populations B22 and Z86. Among these, we selected two of the strongest QTL alleles in each population B22 and Z86 for needle plot analysis to see population wide distribution and effect of exotic and cultivated alleles. The distribution of exotic and cultivated alleles in population B22 is presented in Figure 3, where blue and red bars on *x*-axis represent the BC_2_F_3_ lines carrying cultivated and exotic alleles, respectively. The *y*-axis showed the grain yield in dt/ha. The strongest exotic QTL effect in population showed that the majority of BC_2_F_3_ lines carrying exotic allele were distributed within the high yielding lines. Whereas four lines fall with the low yielding BC_2_F_3_ lines (Figure 3A,B). In population Z86, the distribution of exotic alleles was clearer than B22, where only one line showed an outlier effect exotic allele (Figure 3C,D).

**FIGURE 4:**
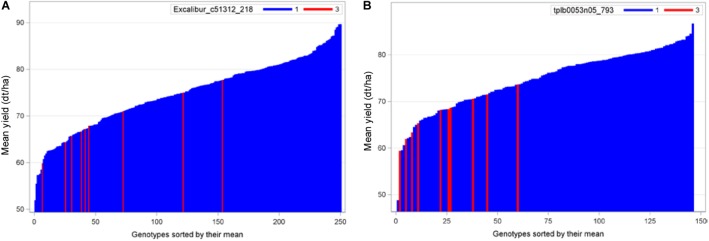
Needle plots of two negative exotic QTL alleles for grain yield in populations B22 **(A)** and Z86 **(B)**. Blue and red bars on *x*-axis represent the BC_2_F_3_ lines carrying homozygous cultivated (CC = 1) and exotic (EE = 3) alleles, respectively. The *y*-axis showed the grain yield in desi tons per hectare (dt/ha).

To compare, we presented the needle plots of two strongest negative exotic QTL alleles for grain yield in populations B22 and Z86. In population B22, the strongest negative exotic QTL alleles was detected at SNP locus Excalibur_c51312_218 (157.9 cM) on chromosome 3D (Figure 4A). Whereas, advanced backcross lines carrying exotic allele at marker locus tplb0053n05_793 (12.3 cM) on chromosome 2D showed decrease in grain yield among the population Z86 (Figure 4B).

## Discussion

The development of hexaploid wheat is one the nature’s wonders by which three different grass species were hybridized. The genetic potential of wheat for yield is reducing due to its lower genetic diversity mainly as results of intensive breeding within the cultivated gene-pool. It has been found that the *A. tauschii* gene-pool (the progenitor of D genome) carried more genetic diversity as compared to the D genome in cultivated varieties (Ogbonnaya et al., 2005; Luo et al., 2017). These reports suggest that a limited gene-pool of *A. tauschii* was involved in the evolution and hybridization of hexaploid wheat. This scenario demands to revisit the genetic potential of *A. tauschii* natural populations to harness the useful variation in cultivated varieties. The issue of D genome diversity and its utility was raised in the past, but no significant efforts were made to enrich the useful genetic diversity at genome level. Till today, the utility *A. tauschii* was largely limited to the incorporation of specific alleles for biotic and abiotic stress tolerance traits (Schachtman et al., 1992; Dubcovsky et al., 1996; Huang et al., 2003; Dreccer et al., 2004a; Naz et al., 2008; Krattinger et al., 2009). The present study was aimed to test the utility of *A. tauschii* for complex traits like yield to add the useful diversity at genome level in the cultivated wheat varieties. For this we employed a population of 400 advanced backcross lines (BC_2_F_3_) carrying chromosomal segments of two exotic synthetic hexaploid wheat accessions in two cultivated wheat backgrounds. The synthetic hexaploid wheat accessions carried the original undomesticated D genome from *A. tauschii* and were established through the production of the amphiploids according to Lange and Jochemsen (1992a,b).

Genome wide SNP marker genotyping showed overall low genetic polymorphism across the D genome as compared to A and B genomes in both populations. These data in line with the characteristic feature of lower genetic diversity across the D genome in cultivated varieties like Chinese Spring (Luo et al., 2017). Recent whole genome sequencing of *A. tauschii* genome also reveals multiple impacts of transposons in determining variation in physical and genetic lengths as well as a faster evolution across the chromosomes of D genome as compared to cultivated wheat (Zhao et al., 2017). Luo et al. (2017) found remarkably more number of gene across the D genome of *A. tauschii* accession as compared cultivar Chinese Spring which suggest a putative chromosome shortening across the D genome of cultivated wheat. These scenarios may be linked with the variation in genetic recombination and abrupt changes (fast evolution) across the chromosomal arms (Akhunov et al., 2003). However, no reports were available yet to describe the extent of chromosome shortening across the D genome of cultivated varieties as compared to *A. tauschii* genotypes. Also, the number and density of SNP was remarkably low in the population B22 as compared to Z86. One major reason behind low marker density in population B22 was the use of 15 k SNP-array instead of 90 k SNP-array in the population Z86. To test this, the population B22 needs to be genotyped with similar 90 k SNP-array chip.

QTL analysis was made by comparing the grain yield data achieved across five environments with the genotype data, to identify useful allele for yield from synthetic wheat accessions especially across the D genome. We employed AB-QTL analysis strategy devised by Tanksley and Nelson (1996) for a straightforward detection and incorporation of the useful exotic alleles back into cultivated varieties to enhance yield and diversity across the D genome simultaneously. No such population has been employed for the improvement of complex traits like grain yield in wheat till today. Our analysis found 20 exotic QTL alleles from synthetic wheat accessions which enhances or at least comparable to the cultivated alleles. The exotic QTL alleles showing high performance relative to cultivated alleles are of great significance for breeding yield, but it is worthy to mention that the exotic alleles showing marginal increase or comparable performance to the cultivated allele are – needed to enrich new genetic diversity without a linkage drag on yield. In the present analysis, the strongest exotic QTL for grain yield were found on chromosomes 1D, 2D, 3D, 5D, and 6D. Bennett et al. (2012) studied grain yield in a doubled haploid population, derived from a cross between RAC875 and Kukri and identified a Kukri allele at Q.Yld.aww-3D for higher yield. This QTL may correspond to the chromosomal position of exotic QTL on chromosome 3D detected in the present study. Earlier Cox et al. (1995) reported the possibility of yield increase in hexaploid wheat using *A. tauschii* backcross populations. Ogbonnaya et al. (2003) evaluated substitution lines under drought stress condition and found substantial variation among the lines where the substitution lines resulted on average 10–41% increase in yield than that of the Australian cultivars. However, the contribution of D genome in this variation remained unclear among the substitution lines. Hitherto, it was suggested that these substitution lines carried beneficial traits like increased capacity for water extraction during critical grain growth phase, vigorous root systems and increased root density relative to the cultivars (Dreccer et al., 2004b). Recently, Bhatta et al. (2018) performed GWAS in a population of synthetic accessions under drought stress conditions and identified 34 genomic regions associated to yield and yield related QTL across the D genome. In the present study, we detected a maximum number of 13 QTL alleles for grain yield across D genome in the population Z86. We found slightly low number of QTL as compared to previous as we employed an advanced backcross BC_2_F_3_ crossing population which does not allow the mapping resolution like execution of GWAS among in natural populations. The numbers of QTL favorable exotic QTL alleles were lower in population B22 as compared to Z86. A reason behind this difference may lie in the resolution of genetic map employed in both populations. Also, the lower density of SNP markers in population B22 may cause lower magnitude of QTL effects as compared to population Z86.

To our knowledge, the present study is the first report that uncovers genome wide favorable exotic allele across the D genome in the cultivated background for their straightforward transfer in elite gene-pool. We are following the established genetic resources in our ongoing work and crossing the strongest exotic QTL alleles with the elite cultivars. From the resulting F1 plants a high resolution double haploid population will be established to refine the marker defined favorable exotic introgressions as well as to test the segregation and effect of additional exotic segments on yield and sustainability traits as *A. tauschii* populations are known for their diversity and adaptive fitness. Further, the established lines can directly be employed in the breeding yield, sustainability as well as enriching D genome diversity of wheat cultivated gene-pool using marker assisted selection and to uncover the role of genes in mediating yield and yield related traits.

## Materials and Methods

### Plant Material

Two advanced backcross populations B22 (250 lines) and Z86 (150 lines) comprising of 400 BC_2_F_3:6_ was established to conduct this research following the advanced backcross strategy of Tanksley and Nelson (1996). The crosses are derived from the two German winter wheat cultivars Batis and Zentos (*T. aestivum* L.) and the two synthetic, hexaploid wheat accessions Syn022 and Syn086 (*Triticum turgidum* spp. *dicoccoides* × *T. tauschii*, Lange and Jochemsen, 1992a,b). AB populations were designated as B22 (Batis x Syn022) and Z86 (Zentos x Syn086). The development of B22 and Z86 until the BC_2_F_3:6_ generation is explained in detail in Kunert et al. (2007).

### Genotyping

The populations B22 (250 BC_2_F_3:6_ lines) and Z86 (150 BC_2_F_3:6_ lines) were genotyped using 15 and 90 k iSelect SNP arrays, respectively. The chromosomal positions of SNP were assigned according to Wang et al. (2014). In addition, a DNA polymorphism survey was conducted between all four parents of the two crosses with a total of 488 SSR markers selected for an even coverage of all three wheat genomes. The chromosomal positions of the SSR markers were obtained from the consensus map of Somers et al. (2004). Percent recombination rate was estimated using the R package R/qtl (Broman et al., 2003).

### Phenotypic Evaluation of Grain Yield

Phenotypic evaluation of grain yield of the BC_2_F_3_ populations B22 and Z86 was carried out under field conditions at five different locations in 2 years (10 environments) across Germany. In each test environment, the AB lines and their recurrent parents were grown in a single randomized block design, containing one plot of each AB line, 20 plots of Batis and 10 plots of Zentos. Net plot sizes (4.5–6.3 m^2^), seed density (310–360 kernels/m^2^) and field management were in accordance with local practice. From each location, grain yield was measured in one-tenth of a ton per hectare calculated from weight of grain harvested per plot and designated as desi ton per hectare (dt/ha).

### QTL Analysis

QTL analysis was carried out with SAS version 9.4 (SAS Institute, 2015). The detection of QTLs was carried out using the mixed hierarchical model:

$$Y_{ijk}=\mu +M_{i}+E_{j}+M_{i}\times E_{j}+\varepsilon_{k(ij),}$$

where μ is the general mean, *M_i_* is the fixed effect of the *i*-th marker genotype, *E_j_* is the random effect of the *j*-th environment, *M_i_* × *E_j_* is the random interaction effect of the *i*-th marker genotype with the *j*-th environment and *ε_l(ij)_* is the error of *Y_ijk_*. At each marker locus only the homozygous genotypes were included in the calculation, because the repeated selfing of heterozygous genotypes leads to a mix of both homozygous genotypes in the derived BC_2_F_3:5_ and BC_2_F_3:6_ field plots, resulting in a false estimate of the performance of true heterozygous genotypes (Pillen et al., 2003). Markers detecting the same significant effect were combined to a single QTL, if linked with ≤20 cM. The relative performance of the homozygous exotic genotype [RP(EE)] was calculated as described by Pillen et al. (2003).

### Circos Diagrams and Needle Plots

The software package Circos was applied to create circular plots with chromosomal ideograms (Krzywinski et al., 2009). The length of the chromosomes and the position of the markers were taken from Wang et al. (2014). For illustration of marker density bins with 5 cM genetic distance were constructed.

For construction of the needle plots for each displayed marker the average phenotypic value of the genotypes containing the synthetic allele or cultivar allele were calculated and plotted against the *y*-axis. The genotypes were sorted according the highest phenotypic value.

## Author Contributions

AN, JL, and KP conceptualized the research. AN, KP, AB, and SD did phenotyping and genotyping of population B22 and Z86. AN, KP, AB, SD, and JL made the data analyses. AN, AB, and SD wrote the manuscript.

## Conflict of Interest Statement

The authors declare that the research was conducted in the absence of any commercial or financial relationships that could be construed as a potential conflict of interest.
